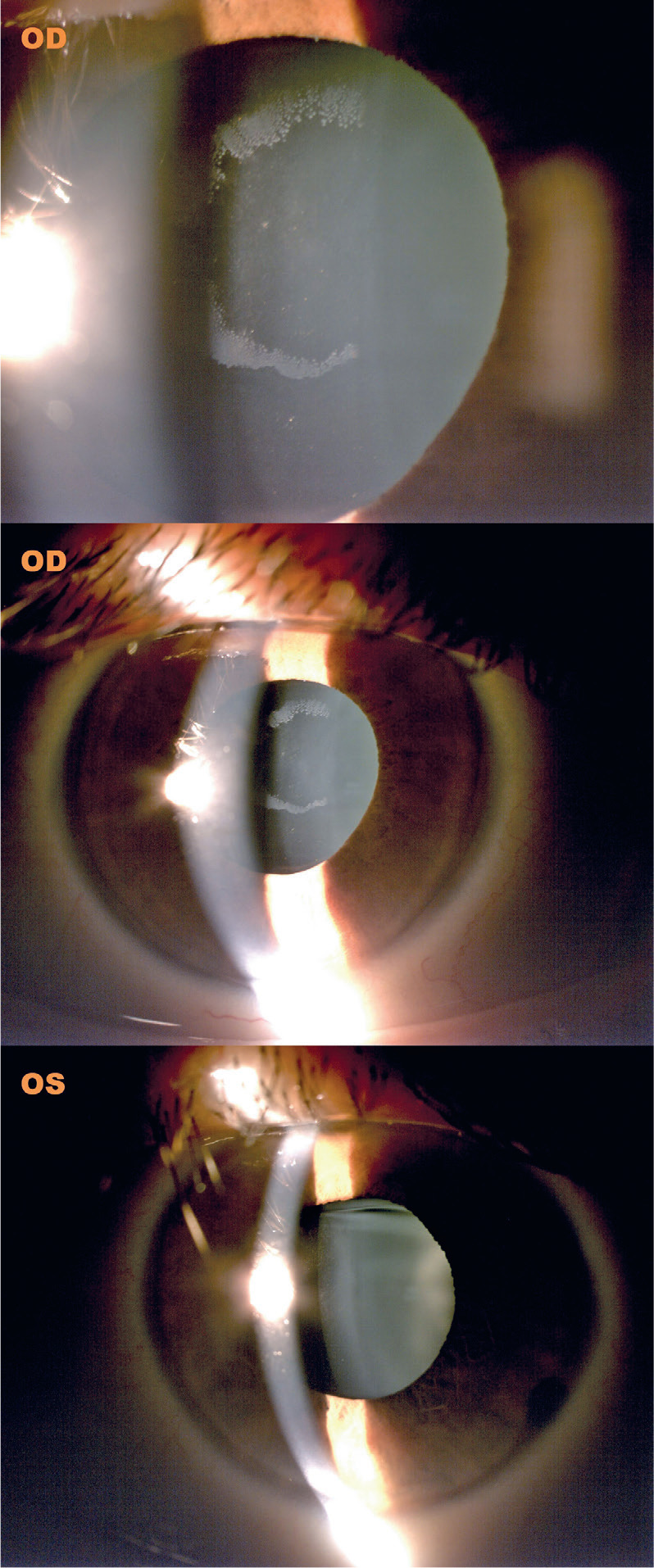# Pseudoexfoliation material deposits in a 43-year-old patient with an implantable collamer lens

**DOI:** 10.5935/0004-2749.2024-0410

**Published:** 2025-06-24

**Authors:** Nikolaos Dervenis, Panagiotis Dervenis

**Affiliations:** 1 Institute of Aging and Chronic Disease, University of Liverpool, Liverpool, United Kingdom; 2 Moorfields Eye Hospital, Moorfields Eye Hospital NHS Foundation Trust, London, United Kingdom

We present a case of pseudoexfoliation material deposits on the implantable collamer lens (ICL) of the right eye in a 43-year-old male patient. The patient had undergone ICL implantation 14 yr prior and presented for a routine follow-up. The implanted ICL was a non-central hole type.

On examination, the patient’s best-corrected visual acuity was 20/20 in both eyes. However, intraocular pressure (IOP) was elevated in the right eye at 26 mmHg, accompanied by increased trabecular meshwork pigmentation. In contrast, the IOP in the left eye was 14 mmHg.

This case represents only the second reported instance of early-onset pseudoexfoliation development associated with an ICL implant, highlighting the need for long-term monitoring in such patients^(1)^.

**Figure:**